# Rabies

**Published:** 1902-03

**Authors:** W. L. Bell

**Affiliations:** Brooklyn, N. Y.


### REPORTS OF CASES.

### RABIES.

By W. L. Bell, D.V.S.,
BROOKLYN, N. Y.

While visiting a call at a stable in Brooklyn, October 31st, I was informed that the owner had just been bitten by a strange dog, and that the S. P. C. A. had been requested to destroy the animal, which was in the basement of the cellar. Upon investigating I found the dog in complete charge of the place, running around in a restless, uneasy manner, no apparent viciousness, but always on the move. I managed to get a noose over his mouth and a collar around his neck, so that I could tie him up. I found out that the dog, a black poodle, had been running in and out of the stable since early morning, and, aside from snapping at some of the horses (which he did not bite), nothing unusual had been noticed. I countermanded the order to destroy him. He had bitten the man in the left thumb and ball of the hand as he reached down to pat the dog's head, making five wounds—two to the bone, the others only cutaneous, and it was only when he was nearly choked that the dog would let go. I was further informed that he had bitten two dogs in the neighborhood, one belonging to Mr. L., the other owner unknown. If his head was touched with a stick or any object he would snap at it, but paid no attention if touched anywhere else. He would try to drink, but could not, but would refuse food of any kind, no matter how tempting. His eyes had a strange look, but not blood-shot; no bark or howl. Paralysis quite noticeable that evening, and he was unable to use his hind legs the next morning, paralysis progressing very rapidly. He died about 2 P. M. that day (November 1st).

I held a hurried post-mortem the next day, and found the subject a French poodle of fairly good breed, about four years old, and in good general condition. He evidently had been at large in the country, as his hair was filled with burrs and dirt. Alimentary canal generally congested ; stomach completely filled with hair and dirt, and contained a canine molar tooth. Small intestines empty, large intestines empty to last part, which had feces of dirt and hairy matter. Bladder full ; kidneys, spleen, and liver macroscopically normal. Both sides of heart empty but for small clot of dark blood. Sent the head and neck to the New York Pasteur Institute for examination that afternoon, November 2d, and was notified, November 4th, that the characteristic rabic lesions were found in the ganglia of the vagus, and that a guinea-pig had been inoculated November 2d, which they reported had died eleven days afterward. Their report is annexed. The fox terrier belonging to Mr. L., which was bitten October 31st, was taken to Dr. McLean's Hospital, November 14th ; and though no history was given of having been bitten by a strange dog, he was such a suspicious case that Dr. McLean had him isolated for observation, and he died three days later of rabies. The man who was bitten started the Pasteur treatment November 5th, six days after having been bitten, and continued it for the usual period of eighteen days. On the afternoon of that day he was compelled to go home, as he suffered from such muscular pains that he could not stand. These pains continued for three days, when the left facial muscles became paralyzed and the muscles of the left upper and lower eyelids, so that when he slept the eye remained open. The physician in charge diagnosed the case as one of neuralgia, and a few days later, when the right facial muscles became paralyzed, said that there had been a slight stroke of paralysis. At this writing, December 9th, the man's condition is improving, so that he can get out of the house ; the eyelid is under control, but the bilateral facial paralysis is still persisting, but not so complete.

It seems to me that this is a case where, had not the man taken the Pasteur treatment, he would have died, before the eighteenth day, of a violent case of rabies, but that the immunity afforded by the preventive inoculation so weakened the rabic condition that the system threw it off, and the man's life will be spared. It hardly seems that a cerebral hemorrhage would affect two identical centres of the brain in different hemispheres, or that a neuralgic condition would give this paralysis.

NEW YORK, November 27, 1901.

DR. W. L. BELL,
BROOKLYN, N. Y.

DEAR DOCTOR: In answer to your request of the 23d inst., we beg to say that the microscopical examination of the vagus ganglia of the dog which you sent us on November 2d revealed the characteristic rabic lesions. This, together with the clinical symptoms which the animal showed, would have been sufficient to establish the diagnosis of rabies. However, the inoculation test was the conclusive proof, the guinea-pig inoculated on November 2d dying in eleven days.

Very truly yours,
NEW YORK PASTEUR INSTITUTE,
PER GEORGE GIBIER RAMBAUD, M.D.

